# Towards elucidating disease-relevant states of neurons and glia by CRISPR-based functional genomics

**DOI:** 10.1186/s13073-022-01134-7

**Published:** 2022-11-18

**Authors:** Kun Leng, Martin Kampmann

**Affiliations:** 1 Institute for Neurodegenerative Diseases, University of California, San Francisco, San Francisco, CA USA; 2 Biomedical Sciences Graduate Program, University of California, San Francisco, San Francisco, CA USA; 3 Medical Scientist Training Program, University of California, San Francisco, San Francisco, CA USA; 4 Department of Biochemistry and Biophysics, University of California, San Francisco, San Francisco, CA USA

## Abstract

Our understanding of neurological diseases has been tremendously enhanced over the past decade by the application of new technologies. Genome-wide association studies have highlighted glial cells as important players in diseases. Single-cell profiling technologies are providing descriptions of disease states of neurons and glia at unprecedented molecular resolution. However, significant gaps remain in our understanding of the mechanisms driving disease-associated cell states, and how these states contribute to disease. These gaps in our understanding can be bridged by CRISPR-based functional genomics, a powerful approach to systematically interrogate gene function. In this review, we will briefly review the current literature on neurological disease-associated cell states and introduce CRISPR-based functional genomics. We discuss how advances in CRISPR-based screens, especially when implemented in the relevant brain cell types or cellular environments, have paved the way towards uncovering mechanisms underlying neurological disease-associated cell states. Finally, we will delineate current challenges and future directions for CRISPR-based functional genomics to further our understanding of neurological diseases and potential therapeutic strategies.

## Background

Advances in next-generation sequencing have enabled the broad application of genome-wide association studies (GWAS) and single-cell profiling studies to neurological diseases. GWAS have uncovered genetic risk factors of disease, and more recently, single-cell or single-nucleus RNA sequencing studies have characterized disease-associated cell states. Both have pointed to cell type-specific contributions to disease, from neurons as well as glia. The next challenge is to uncover the mechanisms driving disease-associated states of neurons and glia and to understand their functional properties. This review will discuss recent developments in CRISPR-based functional genomics that make it possible to systematically elucidate neuronal and glial states relevant to neurological disorders, which will pave the way for targeted manipulation of brain cell states for therapeutic benefit.

## Disease-associated cell states in neurological disorders

### Disease-associated states of glia

Dysfunction of astrocytes and microglia have been implicated in numerous neurodegenerative as well as neuroinflammatory disorders.

The largest GWAS to date of Alzheimer’s disease (AD), the most common cause of dementia, showed that genes associated with AD had significant enrichment of expression in microglia, and in genes related to astrocyte activation [1]; furthermore, AD-associated genetic variants have been linked specifically to enhancers active in microglia [2, 3]. Converging with these insights, single-cell sequencing of mouse models of AD has uncovered disease-associated microglia (DAM) [4–6] and astrocytes (DAA) [7]; both DAM and DAA appear to be driven by amyloid pathology. Beyond mouse models, single-cell profiling of human AD brain tissue has identified a variety of disease-associated perturbations in neurons as well as glia [5, 8–15]. It is likely that in human AD, multiple disease-associated microglial states exist [9, 11], which may partially explain the limited overlap of DAM markers with upregulated genes in microglia found in human AD brain tissue [5].

Regarding Parkinson’s disease (PD), another common cause of dementia, an integrated analysis of GWAS data with expression and methylation data showed that disease-associated genes were overall more prevalently expressed in glia compared to neurons [16]. Consistent with this finding, single-cell sequencing studies of PD have identified the upregulation of inflammatory pathways in reactive astrocytes [17, 18].

In the case of Huntington’s disease, a relatively rarer neurodegenerative disease with Mendelian inheritance driven by repeat expansion in the Huntington gene *HTT*, conditional mouse genetics have pointed towards a role of astrocytes in the initiation and progression of Huntington’s disease [19, 20]. Single-cell sequencing studies of HD have identified reactive astrocytes marked by downregulation of genes involved in glutamate and synapse homeostasis [21, 22], which may contribute to neuron loss via excitotoxicity [23–25].

Beyond neurodegeneration, the significant progress made in our understanding of the autoimmune neuroinflammatory disease multiple sclerosis (MS) through GWAS [26] and single-cell sequencing warrants discussion. Single-cell sequencing studies of MS brain tissue have shed light on how glial cell types are perturbed [27–29], identifying reactive astrocytes with downregulation of genes involved in glutamate and synapse homeostasis [28] or upregulation of inflammatory genes [29], oligodendrocyte subpopulations marked by upregulation of stress response genes [28, 29], and distinct activated microglial states marked by upregulation of genes related to lipid or iron homeostasis [29].

Interestingly, it is worth noting that a common signature of reactive astrocytes found in many of the above studies, which span different disease contexts (AD, PD, HD, MS), appears to be the downregulation of genes involved in glutamate and/or synapse homeostasis [8, 12, 18, 21, 22, 28], suggesting that loss of these homeostatic functions is a common feature of disease-associated reactive astrocyte states.

### Disease-associated states of neurons

Although the contribution of glia to neurological disease is now well-recognized, in many cases the initial driver of disease lies with neuronal dysfunction.

In the case of amyotrophic lateral sclerosis (ALS), which is characterized by degeneration of motor neurons, meta-analysis of GWAS data combined with whole-genome sequencing and expression quantitative trait locus data suggested cell-autonomous disease initiation in glutamatergic neurons involving perturbations in vesicle-mediated transport and autophagy [30]. These findings may also be relevant to frontotemporal dementia (FTD), a related neurodegenerative disease that shares clinical, genetic, and neuropathological features with ALS. Single-cell sequencing studies of FTD have identified dysregulated processes shared across excitatory neurons including endoplasmic reticulum protein processing and autophagy [31]. Beyond neuronal dysfunction, single-cell RNA-seq of FTD has also identified upregulation of interferon-related genes in microglia [32], which may be a consequence of primary neuronal dysfunction.

The primary role of neuronal dysfunction is clear in neurodevelopmental disorders such as autism spectrum disorder (ASD) [28] and schizophrenia [33]. Single-cell sequencing studies of schizophrenia have uncovered perturbations to both excitatory and inhibitory neuron subtypes [34, 35]. Similarly, single-cell sequencing of ASD has shown alterations to synaptic signaling of upper-layer excitatory neurons [36].

### The next challenge: uncovering regulators and functions of disease-associated cell states

From the above sampling of recent studies uncovering disease-associated cell states across various neurological disorders, it is clear that large gaps in our understanding remain: How do disease-associated cell states arise, and what are their roles in disease—either beneficial or detrimental? The next challenge is to gain mechanistic insights into the causes and effects of disease-associated cell states.

**Fig. 1:**
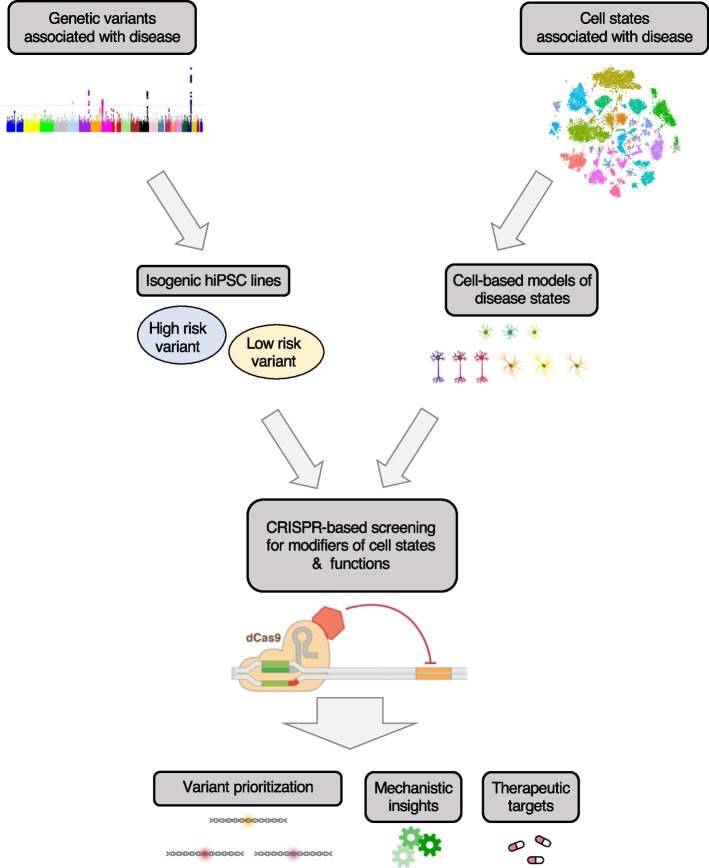
CRISPR-based screens in cell-based models of disease states, in particular those based on hiPSC technology, can provide causal and mechanistic links between genes and disease processes and disease states of neurons and glia, and uncover potential therapeutic targets

To go from correlation to causation, controlled experiments are needed. Recently developed CRISPR-based approaches make it possible to control gene function in relevant brain cell types. The scalability of CRISPR-based perturbations enables genome-wide screens that can systematically identify genes and cellular pathways underlying both normal function of neuron and glia, as well as their disease-relevant states and the associated functional changes (Fig. 1). Such screens can also pinpoint potential therapeutic targets that promote beneficial cellular states or correct pathological cellular dysfunctions (Fig. 1). In the following sections of the review, we will first briefly introduce CRISPR-based functional genomics, then highlight first applications to brain disease and disease-associated cell states, and end with discussing current challenges and future directions.

## Overview of crispr-based functional genomics

CRISPR-based technologies have revolutionized large-scale reverse genetic screening. Methods of CRISPR-based manipulation of gene expression commonly used in genetic screening include direct cutting of DNA with active Cas9 nuclease, which results in gene knockout, or recruitment of transcriptional repressors (CRISPRi) or activators (CRISPRa) through catalytically inactive Cas9, which allows modulation of gene expression levels. More recently, base editing and prime editing through CRISPR-based tools have enabled directly studying the effect of single-nucleotide polymorphisms on cellular phenotypes.

### Gene knockout via Cas9 cutting

Cas9 protein bound to a single-guide RNA (sgRNA) functions as a programmable nuclease [37]. To achieve gene knockout in mammalian cells, DNA breaks can be introduced without providing a repair template, triggering repair through the non-homologous end-joining pathway. Repair errors tend to occur and lead to short deletions [38, 39], which can cause frameshifts in the encoded protein, resulting in complete loss of function.

### Modulation of gene expression via CRISPRi or CRISPRa

A catalytically dead version of Cas9 (dCas9) can be fused to effector domains to recruit them to regulatory sites in mammalian genomes (such as promoters and enhancers) to control gene expression. In an approach called CRISPR interference (CRISPRi), a transcriptional repressor domain (most commonly the KRAB domain) is fused to dCas9 and targeted to transcription start sites to repress gene transcription [40]. CRISPRi can achieve a wide range of reduction in gene expression in human cells, from partial to nearly complete [41], making it suitable for studying the function of essential genes or modeling reduced gene expression in disease (e.g. haploinsufficiency). Compared to RNA interference, an alternative technology for gene knockdown, CRISPRi has substantially fewer off-target effects [40, 41]. CRISPRi can also be used to interrogate the function of distal regulatory elements such as enhancers [42, 43]. A complementary approach is CRISPR activation (CRISPRa), in which dCas9 can be fused to various transcriptional activation domains and targeted to transcription start sites or regulatory elements to promote gene transcription [41, 44–48].

### Base editing and prime editing

Beyond controlling gene expression, CRISPR-based tools have been developed to efficiently modify the endogenous genetic sequence at the single-nucleotide level without a donor DNA template or inducing double-stranded breaks. The first-generation approach, referred to as “base editing,” used dCas9 fused to a cytidine or adenosine deaminase to achieve sgRNA-targeted conversion of G-C base pairs to A-T or A-T base pairs to G-C [49, 50]. Subsequently, a more efficient second-generation approach, referred to as “prime editing,” was developed: a catalytically impaired Cas9 with nickase activity was fused to a reverse transcriptase, and when used in conjunction with an edit-containing RNA template that is a contiguous extension of the sgRNA, all possible single base-pair changes could be programmed [51]. With prime editing, up to 89% of known single-nucleotide variants associated with human disease could in theory be corrected [51], allowing large-scale reverse genetic screens to be performed to study the effect of individual variants [52–55].

### Pooled screening

**Fig. 2:**
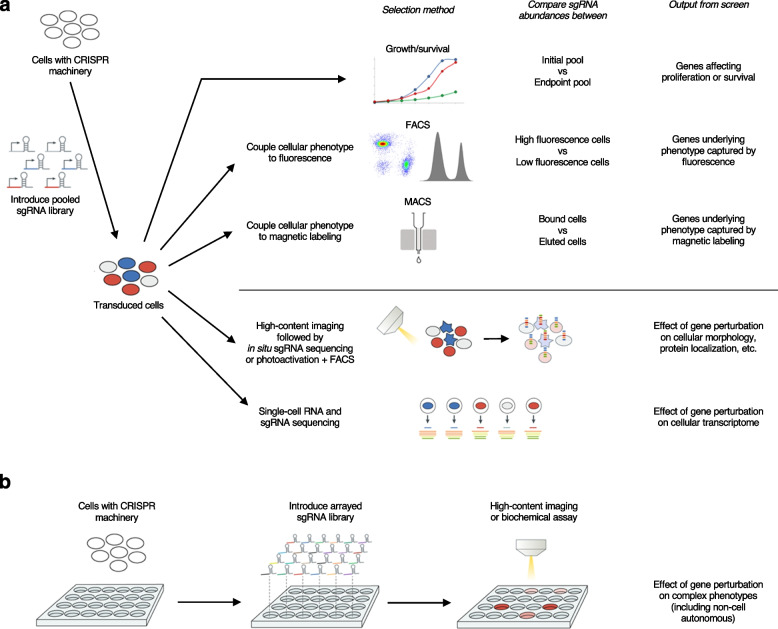
**a** Different approaches to pooled CRISPR-based screening. sgRNA libraries are introduced into a population of cells. Next, subpopulations are selected based on their proliferation/survival or based on physical separation by fluorescence-activated cell sorting (FACS) or magnetic-activated cell sorting (MACS) based on phenotypes of interest, and sgRNA frequencies in different populations are compared by targeted next-generation sequencing. Alternatively, phenotypes of interest are evaluated by microscopy, followed by photoactivation of a fluorescent protein in cells of interest to enable FACS sorting, or by in situ sequencing to identify sgRNAs. Lastly, pooled screens can be read out by single-cell RNA sequencing to identify both the sgRNA expressed in an individual cell and the transcriptomic consequences of gene perturbation. **b** Arrayed CRISPR-based screening. Here, different sgRNAs are individually introduced into cells, enabling additional readouts, including high-content imaging and monitoring of cell non-autonomous phenotypes

CRISPR-based technology can readily be scaled from the perturbation of individual genes to the interrogation of large numbers of genes in genome-wide screens. The most scalable strategy for CRISPR-based screens is pooled screening. Pooled screens typically consist of the delivery of a pooled complex sgRNA library to cells, followed by a selection process based on the cellular phenotype of interest (Fig. 2a). Then, next-generation sequencing is used to monitor changes in the abundance of sgRNAs between cell populations differing in the phenotype of interest, from which genes affecting this phenotype are identified.

A range of cellular phenotypes can be investigated via pooled screening. Cellular survival or proliferation is the simplest phenotype to capture in a pooled screen. In this paradigm, sgRNA libraries are introduced into cultured cells, and samples of the cells are collected at different time points. Quantification of changes in the abundance of individual sgRNAs relative to the initial time point reveals gene perturbations that affect rates of cell proliferation or survival. Pooled survival/proliferation screens can be easily performed at scale and are straightforward to carry out.

To investigate cellular phenotypes beyond proliferation or survival, one approach is to physically separate the cells based on the phenotype of interest, followed by next-generation sequencing of separated cellular populations. Most commonly, this can be achieved by coupling the cellular phenotype of interest to a fluorescence-based readout, separating cells via fluorescence-activated cell sorting (FACS) into bins corresponding to the phenotypes of interest, and then comparing sgRNA abundances across the bins to identify gene perturbations affecting the phenotype of interest. For example, levels of specific proteins can be measured by fluorescently labelled antibodies, and certain cellular activities can be monitored by fluorescent chemical probes or genetically encoded reporters. Compared to survival/proliferation screens, FACS-based screens are more time-consuming to perform, since they are limited by the throughput of FACS. In cases where fluorophore-conjugated reagents can be replaced by magnetic particle-conjugated reagents, magnetic cell sorting (MACS) can be used to physically separate cells [56], which can be less time-consuming than FACS.

Alternatively, if the phenotype of interest can be measured via microscopy, a pooled optical screen can be performed [57, 58]. In a pooled optical screen, sgRNA libraries are first introduced into cells, the cells are then fixed and imaged to capture individual cellular phenotypes, and then the sgRNA expressed in each cell is identified by in situ sequencing. However, pooled optical screens tend to be more time-consuming and smaller-scale given limitations to the speed at which microscopy can be performed at the resolution necessary and the need for iterative rounds of image capture for in situ sequencing. A recent, more scalable approach is the imaging of cells expressing a photo-activatable protein, in which cells with phenotypes of interest are photoactivated and later retrieved physically by FACS for next-generation sequencing [59, 60]. Optical screening approaches make it possible to capture complex multidimensional phenotypes given the rich information that can be obtained via microscopy.

Another approach that can capture complex high-dimensional phenotypes is to couple pooled CRISPR-based gene perturbation to single-cell sequencing [61–64]. Furthermore, single-cell RNA sequencing can be combined with additional readouts to provide “multiomic” phenotypes [65]. However, the main limitation of combining single-cell sequencing with pooled CRISPR-based gene perturbations is the high cost of single-cell sequencing reagents and the cost associated with sequencing, which limits the number of cells that can be profiled and thus the complexity of the sgRNA library.

For all the above pooled screening approaches, parallel screens can be carried out in the presence of experimental perturbations such as drugs, genetic modifiers, or infectious agents, which could give insights into cellular pathways affected by these perturbations.

### Arrayed screening

Despite the efficiency and broad applicability of pooled screens, non-cell-autonomous phenotypes cannot be interrogated in a pooled format: for example, the secretion of factors such as cytokines, neuropeptides, or Aβ peptides from cells. Mechanisms in one cell that elicit a phenotype of interest in a different cell, such as neuronal excitation of target cells or interactions between glia and neurons, are also not accessible via pooled screening.

Phenotypes that cannot be studied in a pooled format can be investigated in an arrayed format, where cells are seeded into multi-well plates and a defined gene is perturbed in each well (Fig. 2b). A multitude of readouts can be obtained from each well, such as high-content imaging, longitudinal live-cell imaging, measurements of electrophysiological activity by calcium or voltage imaging or multi-electrode arrays, quantification of secreted factors, and other biochemical assays.

The main disadvantage of arrayed screening is the logistical challenge of handling large numbers of multi-well plates, generating and delivering arrayed sgRNA libraries, and reading out cellular phenotypes with high throughput—all of which would ideally utilize automated liquid handling and imaging systems. Most academic laboratories do not have the resources to conduct large-scale arrayed screens, but small-scale arrayed screens are an option for secondary screens on a focused set of genes following a large primary pooled screen.

From the brief introduction to CRISPR-based functional genomics above, it is evident that there are many ways to conduct a CRISPR-based screen. Care must be taken to choose a phenotype of interest that is amenable to the screening modality, and once a screening modality is chosen, factors such as scalability and cost must be considered with regard to the library size. For more detailed discussions of the technical considerations in CRISPR-based screening, see the following reviews [66, 67].

## Early applications of CRISPR screens to investigate brain disease

We will first outline some of the early applications of CRISPR-based genetic screening to uncover cellular mechanisms relevant to neurological diseases. Early CRISPR-based screens typically made use of immortalized human cell lines or non-human cells to derive insights into disease-related cellular processes, followed by validation of hits in primary cells, hiPSC-derived cells, or in vivo.

### Modifiers of levels of disease-relevant proteins

Regulators of the cellular levels of specific proteins are of interest in the context of many brain diseases. On the one hand, brain diseases can be caused by the deficiency in a specific protein, and mechanisms that increase the abundance of the protein are potential therapeutic targets. On the other hand, many neurodegenerative diseases are thought to be driven by the toxic aggregation of specific proteins; therefore, a reduction in the level of those proteins may be therapeutically beneficial. Furthermore, the accumulation of toxic protein aggregates or other pathological products in a cell could be associated with disease-associated cell states [68, 69]. Several genetic screens have uncovered positive and negative regulators of the levels of disease-relevant proteins.

Parkinson’s disease is associated with mutations in the gene *PARKIN*, the protein product of which promotes mitophagy. Potting et al. performed a FACS-based genome-wide pooled CRISPR knockout screen in HEK293 cells to identify regulators of PARKIN protein levels, discovering that the transcription factor THAP11 negatively regulates PARKIN expression [70].

Alzheimer’s disease is characterized by intracellular aggregates of the microtubule-binding protein tau. Sanchez et al. performed a FACS-based genome-wide CRISPR knockout screen in SH-SY5Y neuroblastoma cells to identify modulators of tau levels [71]. Chromatin-modifying enzymes, neddylation and ubiquitin pathway members, and components of the mTOR pathway were shown to control tau levels [71]; knockout of TSC1, a critical upstream regulator of the mTOR pathway, was shown to increase tau levels in vivo.

In FTD-ALS, a hexanucleotide (GGGGCC) repeat expansion in the gene *C9orf72* is a common cause of disease. Non-canonical non-AUG (RAN) translation of the repeat region generates toxic dipeptide repeat proteins which are thought to contribute to neuron death [72]. Cheng et al. performed a FACS-based genome-wide CRISPR knockout screen in immortalized retinal pigment epithelial cells (RPE-1) expressing a fluorescent reporter of GGGGCC RAN translation in the glycine-alanine frame and uncovered the RNA helicase DDX3X as a repressor of RAN translation of *C9orf72* hexanucleotide repeat expansions [73].

### Modifiers of the toxicity, aggregation, or uptake of pathology-associated proteins

Cell states of neurons or glia associated with neurodegenerative diseases are thought to be driven by the toxicity, aggregation, or uptake of pathology-associated proteins. The identification of modifiers of the toxicity, aggregation, or uptake of proteins associated with pathology could elucidate the mechanism underlying the emergence of disease-associated cell states, uncover causal determinants of cell type-selective vulnerability, and provide potential neuroprotective therapeutic strategies.

Chen et al. used CRISPRa with randomized sgRNAs in yeast cells to screen for modulators of α-synuclein toxicity in a growth-based screen, identifying genes which when overexpressed protected against α-synuclein toxicity [74]. These genes in yeast mapped to the human genes *PARK7*, *ALS2*, *GGA1*, and *DNAJB1*; overexpression of these genes in human SH-SY5Y neuroblastoma cells also protected against α-synuclein toxicity [74]. See et al. performed a genome-wide FACS-based CRISPRi screen to identify modulators of cytosolic α-synuclein aggregation in HEK293 cells incubated with α-synuclein fibrils, discovering that inhibition of the kinase PIKfyve reduced α-synuclein trafficking from the early endosome to the lysosome, thereby limiting fibril escape from the lysosome and reducing the amount of fibrils that reach cytosolic α-synuclein to induce aggregation [75].

Kramer et al. performed a growth-based genome-wide pooled CRISPR knockout screen in K562 cells to identify modifiers of *C9ORF72*-associated dipeptide repeat protein toxicity, finding genes involved in nucleocytoplasmic transport, the endoplasmic reticulum (ER), proteasome, RNA-processing pathways, and chromatin modification [76]. Knockout of one particular gene *TMX2*, which encodes an ER-resident transmembrane thioredoxin, improved the survival of human hiPSC-derived motor neurons [76].

Rauch et al. performed a CRISPRi screen in H4 neuroglioma cells to find modulators of tau uptake, identifying enzymes in the heparan sulfate proteoglycan biosynthetic pathway as key regulators; 6-O-sulfation was shown to be critical for the interaction of tau with heparan sulfate, which mediates the internalization of tau [77]. The uptake of tau was also found to depend upon the LRP1 receptor, knockdown of which blocked tau spreading in a mouse model [78]. After uptake, tau seeds need to escape the endolysosomal pathway to template aggregation of cytosolic tau. Chen et al. performed a FACS-based CRISPRi screen in HEK293 cells incubated with tau fibrils to find modulators of cytosolic tau aggregation, finding that the ESCRT (endosomal sorting complex required for transport) complex controlled the integrity of the endolysosomal compartment and thereby escape of tau to the cytosol [79]. Duan et al. performed an imaging-based arrayed CRISPR knockout screen in SH-SY5Y cells expressing fluorescently tagged mutant tau protein to identify modulators of tau aggregation induced by addition of exogenous tau fibrils, finding that the NF-κB pathway modulated tau aggregation [80].

Phagocytosis of protein aggregates is thought to contribute to clearance but also play a role in spreading of pathological protein aggregates. Haney et al. performed multiple MACS- or FACS-based genome-wide CRISPR knockout screens in a human myeloid cell line (U937) to find genes modulating the phagocytosis of a variety of phagocytic substrates, uncovering a role for TM2 domain-containing protein 3 (encoded by the AD risk gene TM2D3) in modulating uptake of amyloid beta aggregates [56].

In the span of just a decade, pooled CRISPR-based screens have been used to discover regulators controlling the intracellular concentration, uptake, intercellular spreading, and toxicity of pathological proteins related to neurological diseases, providing insights into cellular processes that may be involved in driving disease-associated cell states of neurons and glia. In many instances, these pioneering studies were able to achieve genome-wide coverage by using immortalized human cell lines which can be easily grown at scale. However, the genetic alterations inherent to immortalized cell lines make it difficult to accurately model the relevant cell type or disease-associated cell states, which is a major limitation of many of the early studies cited above.

## Combining CRISPR and hiPSC technology to dissect disease-associated cell states

In recent years, the maturation of techniques to differentiate human induced pluripotent stem cells (hiPSCs) into various central and peripheral nervous system cell types has greatly expanded our ability to model neurological disease in vitro [81]. A major advantage of using hiPSC-derived cell types is that the effects of pathogenic mutations can be studied in the native context of the human genome and in a cell type-specific manner. To overcome the challenge of variability between different donors, isogenic controls can be generated by CRISPR editing. One approach to generate isogenic controls is to generate hiPSC lines from donors with pathogenic mutations and edit these mutations to the non-disease variants, as exemplified by a large collection of tauopathy-relevant iPSC lines [82]. Alternatively, pathogenic mutations can be introduced into a hiPSC line from a healthy control donor, as exemplified by the NIH hiPSC Neurodegenerative Disease Initiative (iNDI), which aims to model more than 100 mutations associated with Alzheimer’s disease and related dementias in isogenic hiPSC lines [83].

Applying CRISPR-based screening to hiPSC-derived neural cell types holds tremendous potential for elucidating the genetic control of various disease-related processes. As an example, Li et al. performed a growth-based genome-wide pooled CRISPR knockout screen in hiPSC-derived neural progenitor cells to identify modulators of Zika virus infection [84]. Going beyond neural progenitor cells, Guo et al. [85] differentiated hiPSC-derived neural progenitor cells to cortical neurons and performed a survival-based kinome-wide CRISPR knockout screen for regulators of *C9ORF72*-associated poly(PR) dipeptide repeat protein toxicity, identifying and validating NEK6 as an important factor. Notably, the study by Guo et al. required a differentiation process of 70 to 85 days. This demonstrates a key bottleneck for the widespread application of CRISPR-based screening to hiPSC-derived neural cell types, which is the long period of time required to obtain fully differentiated cell types and often heterogeneity in the cell types obtained.

Transcription factor-based fate specification and forward programming of hiPSCs to neural cell types is a promising approach to overcome this obstacle. For example, overexpression of the neurogenic pioneer transcription factor NGN2 from a safe-harbor locus in hiPSCs drives their differentiation into glutamatergic neurons with high efficiency and homogeneity [86]. This enabled the first CRISPR-based screen to be performed in hiPSC-derived neurons by Tian et al. [87], with subsequent expansion to genome-wide coverage [88]. These screens utilized CRISPRi and CRISPRa with survival-based or FACS-based phenotypes to uncover essential genes for survival unique to neurons as well as a novel link between lysosomal failure and ferroptosis in neurons. Knockdown of the lysosomal protein prosaposin in neurons, but not other cell types, resulted in the lysosomal accumulation of lipofuscin, a pathological hallmark of aging and neurodegenerative diseases, which sensitized neurons to oxidative stress by trapping iron [87].

For glial cell types, recent advances in transcription factor-based differentiation have enabled the scalable production of hiPSC-derived microglia [89, 90] and astrocytes [91–93] with high efficiency and homogeneity, enabling the first CRISPR-based screens to be performed in these cell types [89, 92]. Dräger et al. performed CRISPRi and CRISPRa screens with survival-based or FACS-based phenotypes against the druggable genome to identify genes controlling microglial survival, activation, and phagocytosis [89]. Leng et al. also screened against the druggable genome as well as transcription factors to identify genes controlling the response of astrocytes to inflammatory cytokines that drive neuroinflammation using FACS-based phenotypes [92]. In both of these studies, follow-up of hits from the primary screens with CROP-seq, a technique for combining CRISPR-based gene perturbation with single-cell RNA-seq [64], unveiled distinct cell states that overlapped with disease-associated cell states and, also importantly, genes and cellular pathways that drive these cell states.

For example, in Dräger et al. [89], CROP-seq uncovered a range of cell states in the hiPSC-derived microglia at baseline, which align with microglial states observed in microglia isolated from human brains [94] and mouse models of AD [95]. These states include a state marked by high expression of interferon-responsive genes, a state marked by high expression of chemokines, and a state marked by expression of *SPP1* [89]. The interferon-responsive state resembles disease-associated interferon states identified in tauopathies [32, 96] and models of Down syndrome [97], as well as axonal injury and aging [98]. The SPP1 state corresponds to a SPP1 state identified from single-cell RNA-seq of microglia from human AD brain tissue [9, 89]. Knockdown of *MAPK14* increased the abundance of the SPP1 state, whereas knockdown of CSF1R decreased its abundance [89]. Importantly, pharmacological targeting of MAPK14 and CSF1R similarly controlled the frequency of the SPP1 state of microglia [89].

In Leng et al. [92], CROP-seq identified two distinct inflammatory reactive astrocyte states induced by the cytokines IL-1α + TNF + C1q; one inflammatory reactive state was marked by interferon-responsive genes, and the other was marked by acute phase response genes [92]. A large-scale CRISPRi screen uncovered specific regulators controlling which of the two states astrocytes adopted. In particular, STAT3 was found to drive the acute phase response-like cell state and inhibit the interferon-responsive cell state, and this regulatory mechanism was validated in a mouse model of inflammatory astrogliosis [92]. Both states had significant overlap with reactive astrocyte states found in mouse models of neuroinflammation [99, 100], and markers of these states were upregulated in human brain tissue from Alzheimer’s disease and hypoxic-ischemic encephalopathy.

Only in the past several years has the convergence of transcription factor-based hiPSC differentiation protocols and CRISPR-based screening enabled systematic exploration of the genetic control of disease-associated cell states of neurons, astrocytes, and microglia, representing a significant step forward compared to early screens using human cell lines. First applications already demonstrated the potential of CRISPR-based screens in hiPSC models to uncover druggable modifiers of disease-relevant glial states [89, 92], which are potential targets to control glial states for therapeutic benefit.

## Current challenges and future directions

The studies described above highlight the potential of CRISPR-based screens in hiPSC models to elucidate mechanisms driving disease states of neurons and glia, but there are still current technical limitations to overcome as well as limitations inherent to the approach, which we will address in this section.

### Inducible genetic perturbation

For studies employing hiPSC-derived neural cell types for CRISPR-based screening, an important technical consideration is whether genetic perturbations are introduced at the hiPSC stage or later after differentiation. Introducing genetic perturbations at the hiPSC stage is often more straightforward, given that hiPSCs are more efficiently transduced with lentivirus compared to differentiated cells. However, this leads to confounding of the phenotype of interest with the process of differentiation. Introducing the sgRNA library to fully differentiated cells expressing a constitutive Cas9 or CRISPRi/a system would circumvent this problem, but not all cell types can be efficiently transduced with lentivirus (e.g., microglia). Alternatively, inducible CRISPRi/a systems, such as the ones established in Draeger et al. [89] and Tian et al. [87], can be used; however, the degree of gene knockdown with inducible CRISPRi is lower in some instances [87, 89]. Further optimization of inducible CRISPRi systems may overcome this limitation.

### Modeling aging in vitro

A technical challenge that is inherent to the approach of using hiPSC-derived neural cell types for CRISPR-based screening is the fact that the reprogramming of somatic cells to hiPSCs erases the chromatin alterations associated with aging [101] and that differentiated cells derived from hiPSCs resemble those found in fetal development [102]. These properties are desirable for modeling developmental diseases such as ASD and schizophrenia, but not so much for diseases of aging such as dementia. Several strategies have been proposed to model aging in iPSC-derived cells [103, 104], but it remains to be seen which aspects of aging are recapitulated. An alternative is the “direct” reprogramming of somatic cells, such as fibroblasts, from aged donors to the desired neural cell type, such as neurons [105, 106], which preserves some of the chromatin alterations and cellular hallmarks associated with aging. Although it would be difficult to achieve direct reprogramming on a scale sufficient for genome-scale CRISPR-based screening, it could be a useful approach for validating whether hits from a primary screen in hiPSC-derived cells validate in “aged” cells.

### From in vitro to in vivo

Disease-related cell states and neuron-glia interactions rely on the complex interactions between cell types and their environment, which can only partially be modeled in vitro.

One approach to overcome this limitation is to make the in vitro environment more similar to the in vivo environment. For example, hiPSC-derived brain cell types can be assembled into multilineage assembloids which capture some of the complex interactions among neurons and glia that occur in three-dimensional space [107–109]. In the field of cancer, comparison of CRISPR screens in two-dimensional monolayers vs. three-dimensional spheroids has already shown crucial differences, with genes having differential phenotypes between 2D cultures and 3D spheroids being enriched for cellular pathways underlying cancer dependencies [110].

Compared to multilineage assembloids, cerebral brain organoids offer an even higher level of resemblance to the in vivo environment and have been used to model important disease-related processes in AD [111], PD [112, 113], ALS/FTD [114, 115], and numerous other diseases. Impressively, Esk et al. performed a targeted CRISPR-based screen against candidate microcephaly-related genes in cerebral organoids and identified a role of endoplasmic reticulum dysfunction in the pathogenesis of microcephaly [116]. Paulsen et al. generated cerebral organoids haploinsufficient in ASD risk genes and characterized cell type-specific developmental abnormalities with single-cell RNA-seq, finding that haploinsufficiency of *KMT5B*, *PTEN*, and *CHD8* accelerated the development of cortical neurons [117].

Besides increasing the similarity of in vitro models to the in vivo environment, another approach is to perform screens directly in vivo. A small number of pioneering studies have been published so far utilizing in vivo CRISPR-based screening to study brain diseases and biology. Wertz et al. performed a genome-wide CRISPR knockout screen in brains of wild-type vs. mutant HTT mice via injection of lentiviral sgRNA libraries, identifying genes that modify neuronal survival and mutant HTT toxicity [118]. Ruetz et al. performed a primary in vitro genome-wide screen to identify regulators of neural stem cell proliferation and then followed up with a targeted screen against the top hits in vivo, validating 23 genes which when knocked out boosted neural stem cell proliferation and the production of new neurons in vivo [119]. Xin et al. combined single-cell RNA-seq with CRISPR-based gene perturbations to the in vivo setting via electroporation of sgRNA libraries to the brains of mouse embryos with constitutive Cas9 expression, identifying neuronal and glial abnormalities associated with loss of function in autism risk genes [120].

An exciting extension of in vivo CRISPR-based screening that has yet to see published studies would be the transplantation of human hiPSC-derived glial cell types into the brains of control vs. disease model mice [91, 121–124] to study processes controlling glial activation and their response to pathological CNS environments.

### Connecting to human genetics and disease

Almost all the studies cited so far have examined the effect of gene knockout or knockdown in a “wild-type” genetic background, which in practice means a genetic background consisting of unknown variants compared to the population reference. An exciting next step will be to perform parallel screens in differentiated cells derived from hiPSCs harboring pathogenic mutations vs. isogenic control hiPSCs, which could reveal genes controlling the disease phenotype in general vs. genes specifically affecting processes perturbed by the pathogenic mutation. This would be particularly applicable to familial mutations, or other genetic variants with strong effect sizes, such as variants in APOE.

A different approach is likely needed to study variants with small effect sizes, which compose the vast majority of variants identified via GWAS. Recently, Cooper et al. [125] used a pooled CRISPRi screen in combination with single-cell RNA-seq to validate the gene-regulatory activity of 42 non-coding variants in hiPSC-derived neurons and microglia. This approach enabled the identification of regulatory target genes, and the cell type specificity of the regulatory elements (several of which were only active in microglia but not in neurons, or vice versa). The single-cell RNA-seq readout can also reveal the effect of regulatory genetic variants on cell states.

Alternatively, prime editing or base editing could be used in future studies in brain-relevant cell types to investigate the effect of single-nucleotide variants on a disease phenotype in pooled screens, which may aid in the functional annotation and prioritization of variants of unknown significance often identified in clinical whole-exome or whole-genome sequencing.

## Conclusions

CRISPR-based screening has already yielded important insights into the mechanisms of neurological and neurodevelopmental diseases and holds great promise for uncovering the cellular pathways that drive disease-associated cell states. Future applications of CRISPR-based screening to multilineage assemblies, cerebral brain organoids, and the in vivo setting will further close the gap between our descriptive knowledge of disease-associated cell states and genetic variants associated with disease by uncovering causal molecular mechanisms connecting the two. Such a mechanistic understanding will likely inform the development of novel therapeutic strategies for these devastating diseases. In addition, applying CRISPR-based screening to study the effect of single-nucleotide polymorphisms contribute to the functional annotation and prioritization of variants of unknown significance frequently found in clinical whole-exome or whole-genome sequencing, which will aid in the diagnosis of genetic disorders.

## Data Availability

Not applicable.
